# Novel *EPG5* Mutation Associated with Vici Syndrome Gene

**DOI:** 10.1155/2022/5452944

**Published:** 2022-07-05

**Authors:** Frouzandeh Mahjoubi, Samira Shabani, Sogand Khakbazpour, Aylar Khaligh Akhlaghi

**Affiliations:** ^1^Department of Clinical Genetic, National Institute of Genetic Engineering and Biotechnology (NIGEB), Tehran, Iran; ^2^Solaleh Medical Genetic Lan, Tehran, Iran; ^3^The Islamic Azad University, Shahr-e-Qods Branch, Tehran, Iran

## Abstract

**Introduction:**

Vici syndrome (also known as immunodeficiency with cleft lip/palate, cataract, and hypopigmentation and absent corpus callosum) is considered as a progressive neurodevelopmental multisystem disorder. Till date, only 80 cases, including our patient, with this syndrome have been reported .This syndrome is characterized by agenesis of the corpus callosum, hypopigmentation of the eyes and hair, cataract, cardiomyopathy, combined immunodeficiency, hearing loss, seizures, and additional multisystem involvements which have been reported as case reports in the past. *Clinical Manifestation*. A 5-year-old girl, who is a product of consanguineous marriage, was referred to our center with developmental delay, optic atrophy, blindness, spasticity, seizure, movement disability, and spasticity. Her magnetic resonance imaging (MRI) test showed agenesis of the corpus callosum and her metabolic test reported normal.

**Materials and Methods:**

In our laboratory, blood sample was obtained from the patient. DNA was extracted from lymphocytes, and whole exome sequencing (WES) using next generation Illumina sequencing was performed.

**Result:**

A novel (private), homozygous, nonsynonymous mutation c.A3206G (p.Y1069C Het) in *EPG5* gene was detected; in continuum, testing for this specific variant in her parents was carried out. DNA sequencing of the PCR-amplified product of the *EPG5* exon 17 showed that her parents were heterozygote for this variant. These mutations have not been reported before and therefore classified as variation of unknown significance (VUS). Mutation in this gene is shown to cause autosomal recessive Vici syndrome.

**Conclusion:**

Since clinical features of Vici syndrome has overlap, its diagnosis is differential and developmental delay occurs in 98% of reported cases. Vici syndrome can be considered as one of the main causes of developmental delay, and this syndrome can be introduced as a novel group of inherited neurometabolic conditions and congenital disorders.

## 1. Introduction

Vici syndrome (also known as immunodeficiency with cleft lip/palate, cataract, and hypopigmentation and absent corpus callosum, Dionisi Vici Sabetta Gambarara syndrome) is considered as a progressive neurodevelopmental multisystem disorder [1]. This syndrome is characterized by agenesis of the corpus callosum, hypopigmentation of the eyes and hair, cataracts, cardiomyopathy, combined immunodeficiency, hearing loss, seizures and additional multisystem involvements which have been reported as case reports in the past [2]. Vici syndrome is caused by recessive mutations in *EPG5* gene (OMIM #615068). which encodes one of the main regulators of the autophagy pathway [2, 3]; therefore, it seems mutations in the *EPG5* gene underling the autophagy pathway by blocking the autophagosome-lysosome fusion mechanism [3]. Using the next generation technique, we introduce a 5-year-old girl as a new case of Vici syndrome with a new mutation in *EPG5* gene.

## 2. Case Report

**Figure 1:**
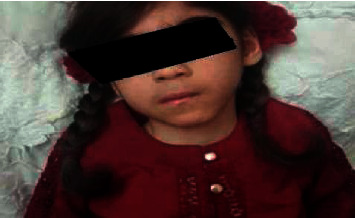
A 5-year-old girl with Vici syndrome.

A 5-year-old girl, who is a product of consanguineous marriage, was referred to our center with developmental delay, optic atrophy, blindness, seizure, movement disability, and spasticity. Her MRI test showed agenesis of the corpus callosum and her metabolic test reported normal (Figure 1).

**Figure 2:**
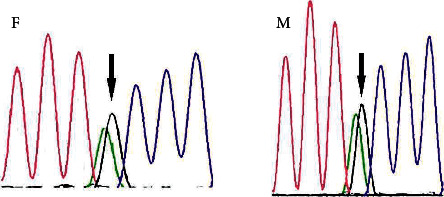
Heterozygous mutation c.A3206G (p.Y1069C; Het) on *EPG5* gene has been detected in father (F) and in mother (M) of this case (5-year-old girl) by DNA sequencing of the PCR-amplified product of the *EPG5* exon 17. Therefore, her parents are the carrier of this mutation. Since the parent found to be the carrier of the above mutation, the patient is very likely affected with Vici syndrome. Heterozygous mutation c.A3206G (p.Y1069C; Het) on *EPG5* gene has been detected in the 5-year-old girl.

In our laboratory, blood sample was obtained from the patient. DNA was extracted from lymphocytes, and then, whole exome sequencing (WES) using next generation Illumina sequencing was performed. Two novel (private), homozygous, nonsynonymous mutations in *EPG5* gene were detected (Table 1). In continuum, testing for this specific variant in her parents was carried out. DNA sequencing of the PCR-amplified product of the *EPG5* exon 17 showed that her parents were heterozygote for this variant (Figure 2).

These mutations have not been reported before and therefore classified as variant of unknown significance (VUS). Mutation in this gene is shown to cause autosomal recessive Vici syndrome. Since the parents were found to be the carriers of the above mutation, the patient is very likely affected with Vici syndrome.

## 3. Discussion

Here, we describe an Iranian patient with Vici syndrome, born out of consanguineous marriage in a family from Persian descent. We report a novel homozygous, nonsynonymous mutation c.A3206G (p.Y1069C Het) in *EPG5* gene. This case had developmental delay, optic atrophy, blindness, spasticity, seizure, movement disability, and spasticity. Her MRI test showed agenesis of the corpus callosum and her metabolic test reported normal.

Vici syndrome was first report in 1988 by Carlo Dionisi-Vici et al. (Rome, Italy) [7, 8, 10]. Since then, only a few articles in the literature were reported with the name of Vici syndrome [2]. Subsequently, only about 80 people including our patient were reported with this disease up to date 5−9. This syndrome in most of the reported cases was related with a number of typical features including agenesis of the corpus callosum (97.4%), developmental delay (97.4%), recurrent infections (98.73%), immunodeficiency (75.94%), cardiomyopathy (65%), abnormal EEG (80–99% patients), intellectual disability (80–99% patients), cataracts (75%), seizures (65%), and renal abnormalities (15%) [6 −11]. The clinical diagnostic method for Vici syndrome comprised of MRI of the brain, ophthalmic examination, EEG, cardiac ECHO, and sequencing of *EPG5* to confirm the diagnosis (https://www.malacards.org/card/vici_syndrome).

In 2013, it was reported that mutations in *EPG5* have been associated with Vici syndrome [6 −11]. Ectopic P granules protein 5 homolog (*EPG5*, OMIM # 615068) is involved in the autophagy pathway and encodes one of the important regulators of this pathway. Autophagy is a highly conserved cellular degradative process which is involved in *n* removal of organelles and defective proteins, and it is essential for removing waste products and reducing cell consumption [12].

*EPG5* gene contains 47 exons, located on chromosome 18q21.1, has a length of 11967Kb (NC_000018.10), and coding 2579 amino acids (NP_066015.2) with a molecular weight of 280 kDa [7]. *EPG5* encodes a large coiled coil domain-containing protein involved in the formation of lysosomes and functions in autophagy during starvation conditions (https://www.ncbi.nlm.nih.gov/gene/57724#gene-expression). It appears as mutations in *EPG5* gene blocks the autophagosome-lysosome fusion mechanism [3]. This gene has 1 isoform, and its protein expresses in most tissues, especially the bone marrow and thyroid tissues (https://www.ncbi.nlm.nih.gov/gene/57724#gene-expression).

Nearly 42 *EPG5* mutations have been reported to date for this syndrome, the majority of them truncating and private to distinct families [13]. Thomas et al. in 2013 carried out exome and Sanger sequence analysis in some affected Vici syndrome patients, and they found that recessive mutations in *EPG5* was associated with this disorder [6]. Since then, different mutations of *EPG5* gene were reported in which most of them are private [14].

There is no specific treatment for this syndrome yet and therapeutic interventions are more supportive to increase survival and relieve symptoms, but we hope a better understanding of the autophagy pathway will help the improvement of targeted therapies in future.

## 4. Conclusion

At first, the case features were suspected for metabolic disorders which were excluded since all of them were negative for this girl. While Vici syndrome is related with many different symptoms in metabolic diseases and congenital defects, we recommend that this syndrome should be investigated for in any case with agenesis of the corpus callosum if mitochondrial, glycogen, or lysosomal storage disorders have been excluded.

## Figures and Tables

**Table 1:** Two novels (private), homozygous, missense, and nonframeshift insertion mutations in *EPG5* gene detected by whole exome sequencing (WES) using next generation Illumina sequencing.

Chr	Start	Ref	Alt	Gene	Zygosity	Function	Detail
Chr18	45917712	T	C	*EPG5*	Homo	Nonsynonymous	*EPG5*: NM-020964: exon17: c.A3206G: p.Y1069C
Chr18	45967193	—	TGGCCT	*EPG5*	Homo	Nonframeshit insertion	*EPG5*: NM-020964: exon1: c.46-47insAGGCCA: p.S16delinsKAS
SIFT	PolyPhen2	MutTaster	Pred		BayanGene	1000GenomeFreq	OMIM
0.01	1	1	D	CADD-Phred	2	.	Vici syndrome
.	0	.	.	.	2	0.0	Vici syndrome
